# New Species and Records of *Nothofagicola* n. sp. (Eriophyoidea, Phytoptidae) from Chile and Updated Key to the World Genera of the Tribe Sierraphytoptini Keifer 1944

**DOI:** 10.3390/ani16081246

**Published:** 2026-04-18

**Authors:** Philipp E. Chetverikov, Lourdes E. Peralta Alba

**Affiliations:** 1 Zoological Institute, Russian Academy of Sciences, Universitetskaya nab., 1, 199034 St. Petersburg, Russia; 2 Laboratorio Entomologia y Acarologia Region del Maule, Servicio de Agricultura, Carmen 560, 3e piso, Curicó 3340001, Chile; lourdes.peralta@sag.gob.cl

**Keywords:** relict, endemic, vagrant, mites, phytophagy, systematics

## Abstract

The monophyletic lineage Phytoptidae s. str. (Acariformes, Eriophyoidea) comprises ~75 described species in 17 genera associated with angiosperms. Since the last keys to the world genera of Eriophyoidea were published in 2003, five new phytoptid genera have been described: *Neoprothrix* (Reis & Navia 2014), *Borassia* Chetverikov, Craemer 2017, *Solenoplatilobus* Chetverikov & Craemer 2016, *Solenocristus* Chetverikov et al. 2018, and *Calventer* Chetverikov 2025. Here, we provide an updated generic key for the tribe Sierraphytoptini (Phytoptidae), describe a new genus *Nothofagicola*, propose a new combination *N. nothofagalis* **n. comb.** (Chetverikov et al. 2018) (transferred from *Solenocristus*), report the first findings of *N.* cf *nothofagalis* from *Nothofagus pumilio* and *No. antarctica* from Southern Chile and describe three new species of *Nothofagicola* collected in Central Chile from stems of three *Nothofagus* species (Fagales: Nothofagaceae): *N. alpinae* **n. sp.** from *No. alpina*, *N. bicristata* **n. sp.** from *No. alessandrii*, and *N. licanteniensis* **n. sp.** from *No. glauca*. We also provide a corrigendum to our previous paper and state that the correct accession numbers for mitogenomic sequences of *Leipothrix aegopodiae* and *L.* cf *knautiae* are OR268621 and OR268622, respectively (vice versa in the paper).

## Simple Summary

Gall mites are tiny phytoparasites that can cause growth deformities on plants, induce gallogenesis, and transmit viruses. Within this group, mites of the family Phytoptidae (phytoptids) represent a distinct lineage, often associated with relict and endemic plants such as southern beeches (*Nothofagus*). In this study, we focus on a group of phytoptids found on *Nothofagus* trees in South America. We describe a new genus of the tribe Sierraphytoptini, *Nothofagicola*, transfer one previously described species to this genus, and describe three new *Nothofagicola* species collected from different *Nothofagus* hosts in Central Chile. Additionally, we provide an updated identification key for the tribe Sierraphytoptini, as five new phytoptid genera have been described since the last comprehensive key was published in 2003. Finally, we correct an error from our previous publications regarding DNA sequence accession numbers. This work expands our knowledge of the diversity and host associations of phytoptid mites in the Southern Hemisphere.

## 1. Introduction

Acariform mites of the superfamily Eriophyoidea are an ancient lineage of permanent parasites of vascular plants, sister to soil mites of the family Nematalycidae, and originated ~376 (314–441) Ma [1]. They include four early-diverged lineages: Pentasetacidae (associated with *Araucaria* and *Cupressus*), Phytoptidae s. str. (associated with angiosperms), Nalepellidae (associated with gymnosperms), and Eriophyidae s. l. (associated with ferns, gymnosperms and angiosperms) [2]. In the most recent generic key of Eriophyoidea [3], the members of lineages Pentasetacidae, Phytoptidae s. str. and Nalepellidae are included in a putatively paraphyletic family, Phytoptidae s.l., defined by a set of plesiomorphies (e.g., elongated spermathecal tubes and retention of tibial solenidion *φ* on leg I as well as setae *vi*, *ve*, and *c1*) observed in various combinations in different phytoptid taxa.

Phytoptidae s. str. comprises ~75 known species inhabiting angiosperms, with the highest generic diversity observed in commelinids (monocots) and superrosids (eudicots) [4,5,6]. Molecular phylogenetics inferred the monophyly of Phytoptidae s. str., suggested no basal codivergence of phytoptids with angiosperms, and inferred the paraphyly of the phytoptids associated with monocots and eudicots. Since the last keys to the world genera of Eriophyoidea were published in 2003 [3], five new phytoptid genera have been described: *Neoprothrix* (Reis & Navia 2014), *Borassia* Chetverikov, Craemer 2017, *Solenoplatilobus* Chetverikov & Craemer 2016, *Solenocristus* Chetverikov et al. 2018, and *Calventer* Chetverikov 2025. Currently the family Phytoptidae s. str. includes 17 genera nearly equally distributed among monocot (*Acathrix*, *Borassia*, *Calventer*, *Mackiella*, *Novophytoptus*, *Oziella*, *Propilus*, and *Retracrus*,) and eudicot (*Austracus*, *Fragariocoptes*, *Neopropilus*, *Nothofagicola* **n. g.**, *Phytoptus*, *Prothrix*, *Sierraphytoptus*, *Solenocristus*, and *Solenoplatilobus*) hosts.

Seven phytoptid genera (*Acathrix*, *Austracus*, *Oziella*, *Nothofagicola* **n. g.**, *Solenocristus*, *Solenoplatilobus*, and species of the group I of *Phytoptus* [7]) possess identical chaetom consisting of 64 setae: four setae on the prodorsal shield (paired *ve* and *sc*) and complete plesiomorphic sets of leg setae (including tibial solenidia *φ* I) and gnathosomal and opisthosomal setae (including paired *c1* on the dorsal opisthosoma) [6,8]. Formally, these genera are assigned to different subfamilies differing in relative width and number of ventral and dorsal opisthosomal annuli: Phytoptinae have approximately equal number of similarly wide dorsal and ventral annuli (*Oziella*, *Acathrix*, *Phytoptus*) and Sierraphytoptinae have wider and fewer dorsal annuli (Sierraphytoptini: *Austracus*, *Solenocristus*, *Solenoplatilobus*) [3]. Molecular phylogenetics revealed the polyphyly of these subfamilies [4], and most probably they will be reorganized in the future.

Besides plesiomorphic morphology, many phytoptid taxa are associated with relict and endemic host plants, reinforcing the relictual nature of Phytoptidae s. str. [9]. For instance, most members of *Phytoptus* described by H. Keifer from North America live on Californian endemics, and species of the genera *Solenocristus* and *Austracus* are associated with the ancient plant genus *Nothofagus*. This is a Gondwanian plant taxon that in previous epochs was distributed across continents in the Southern Hemisphere, including Antarctica, and currently exhibits a disjunctive distribution in South America and Australasia [10]. Up to now, phytoptids have been recorded only on South American species of *Nothofagus* [5,11]. Besides phytoptids, only members of the family Eriophyidae have also been registered on *Nothofagus* spp. in New Zealand and South America [12,13,14,15].

The phytoptid genus *Solenocristus* Chetverikov et al. 2018 was described based on material collected from endemic South African eudicot plant species of the genera *Schotia* and *Searsia* [16]. Morphologically *Solenocristus* is very distinctive, since it is the only sierraphytoptine genus possessing a median opisthosomal ridge. Later, a new species, *S. nothofagalis* Chetverikov et al. 2020, was described from *Nothofagus pumilio* from southern Argentina [17]. It was provisionally assigned to *Solenocristus* because, despite its great similarity with *Solenocristus* spp., it did not have the median ridge. The authors noted that the systematic position of *S. nothofagalis* needs further testing, since they assumed that, similar to members of the family Eriophyidae [3], phytoptid mites differing in the presence/absence of the median opisthosomal ridge may belong to different lineages and represent separate genera.

In 2025 in Chile, we collected three new phytoptid species that, similar to *S. nothofagalis*, did not have the median opisthosomal ridge. In this paper we establish a new genus, *Nothofagicola* **n. g.**, to accommodate this group of non-*Solenocristus* species associated with *Nothofagus*, describe three new species of *Nothofagicola* from Central Chile, transfer *S. nothofagalis* to this genus, and provide an updated key to the world genera of the tribe Sierraphytoptini Keifer 1944.

## 2. Materials and Methods

**Morphology.** Fresh plant material (twigs of *Nothofagus* ~20–30 cm long) was collected in February and March 2025 in Chile. The morphological identification of *Nothofagus* was performed based on the key by Gandolfo & Romero [18]. Eriophyoid mites were collected from leaves and young stems using a fine minuten pin and a dissecting microscope and were kept in Eppendorf tubes filled with 96% ethanol in a freezer (−20 °C). The mites from the ethanol material were investigated at the Zoological Institute of the Russian Academy of Sciences (ZIN RAS) in Saint-Petersburg, Russia. These mites were mounted in modified Berlese medium with iodine [19]. They were then cleared on a heating block at 90° C for ~5 h. The slide-mounted specimens were examined with differential interference contrast light microscopy (DIC LM) and phase contrast light microscopy (PC LM). In the description of the new species, the measurements of the holotype (female) are given in addition to ranges of paratype females. All length-based measurements are given in micrometers (μm) except when mentioned otherwise. Asterisks (*) denote measurements with no variation. Classification and terminology of eriophyoid morphology follow that reported in previous papers [3,7,8,20]. To illustrate morphological differences between sierraphytoptine genera, we used new DIC LM photographs obtained in this study, as well as unpublished confocal laser scanning microscopy (CLSM) images from our personal archives that were acquired using a previously described methodology [7]. 

**DNA extraction, PCR and sequencing.** One to three mite specimens were used for DNA extraction. They were crushed separately by a sterile fine pin in a 2 μL drop of distilled water on a cavity well microscope slide. DNA of the mites was extracted, and fragments of two marker genes (*COI* and *D1D2 28S*) were amplified and sequenced using previously specified protocols and equipment [21].

## 3. Results

### 3.1. Systematic Remarks on Eriophyoid Mites from the Tribe Sierraphytoptini Keifer 1944

**Family Phytoptidae Murray 1877**

**Subfamily Sierraphytoptinae Keifer 1944**

**Tribe Sierraphytoptini Keifer 1944**

**Diagnosis**. Phytoptid mites with dorso-ventrally differentiated opisthosomal annuli; lacking internal vertical seta *vi* and retaining dorsal opisthosomal seta *c1*.

**Remarks**. Currently this tribe comprises 16 species and nine genera: *Austracus* Keifr 1944 (1 species), *Fragariocoptes* Roivainen 1951 (4 spp.), *Neopropilus* Huang 1992 (1 sp.), *Neoprothrix* (Reis & Navia 2014) (1 sp.), *Nothofagicola* n. g. (4 spp.), *Prothrix* Keifer 1965 (1 sp.), *Solenoplatilobus* Chetverikov & Craemer 2016 (1 sp.), *Solenocristus* Chetverikov et al. 2018 (2 spp.), and *Sierraphytoptus* Keifer 1939 (1 sp.). The most distinct morphological characters separating sierraphytoptine genera include topography of dorsal opisthosoma, position of the tubercles of setae *sc* and *ve* on the prodorsal shield and presence/absence of telosomal pseudotagma [22], opisthosomal setae *c2*, *d*, *e* and tibial solenidion *φ* I and setae *l*’ I (Table 1).

**Updated Key to the world genera of the tribe Sierraphytoptini Keifer 1944**
  **1.** Opisthosomal setae *c1* present------------------------**2** (**Tribe Sierraphytoptini Keifer 1944**)
  —Opisthosomal setae *c1* absent------------------------------------**Tribe Mackiellini Keifer 1946**
  **2.** Tibial solenidion *φ* I present----------------------------------------------------------------------------**3**
  **—**Tibial solenidion *φ* I absent-----------------------------------------------------------------------------**9**
  **3.** Opisthosomal setae *d* and *e* and tibial seta *l*’ I present, telosomal pseudotagma absent----------------------------------------------------------------------------------------------------------------------**4**
  **—**Opisthosomal setae *d* and *e* and tibial seta *l*’ I absent, telosomal pseudotagma present----------------------------------------------------------------------------------------------------------------------**7**
  **4.** Frontal lobe of prodorsal shield present; tubercles of *ve* displaced below the anterior margin of prodorsal shield, opisthosoma compact, fusiform--------------------------------------**5**
  **—**Frontal lobe absent; tubercles of *ve* not displaced below the anterior margin of prodorsal shield; opisthosoma more wormlike, elongated--------------------------***Austracus* Keifer 1944**
  **5.** Dorsal opisthosomal annuli forming distinct medial ridge----------------------------------------------------------------------------------------------------------***Solenocristus* Chetverikov et al. 2016**
  **—**Dorsal opisthosoma flattened, without medial longitudinal ridge----------------------------**6**
  **6.** Prodorsal shield with thin broad frontal lobe covering basal chelicerae, dorsal annuli narrow and microtuberculated---------------***Solenoplatilobus* Chetverikov & Craemer 2016**
  **—**Prodorsal shield with thicker subtriangular frontal lobe, dorsal annuli notably wider than opisthosomal annuli, overlapping and devoid of microtubercles, associated with Nothofagaceae------------------------------------------------------------------------***Nothofagicola* n. g.**
  **7.** Setae *sc* present, tubercles of *sc* displaced to anterior part of prodorsal shield--------------------------------------------------------------------------------------------------------***Prothrix* Keifer 1965**
  **—**Setae *sc* absent----------------------------------------------------------------------------------------------**8**
  **8.** Opisthosomal setae *c2* present--------------------------------***Neoprothrix* (Reis & Navia 2014)**
  **—**Opisthosomal setae *c2* absent-------------------------------------------***Neopropilus* Huang 1992**
  **9.** Opisthosoma with two lateral longitudinal ridges and concave furrow between them; rear prodorsal shield margin distinct; tubercles of *sc* situated in deep indentation of posterior edge of prodorsal shield. Associated with Betulaceae------------------------------------------------------------------------------------------------------------------------***Sierraphytoptus* Keifer 1939**
  **—**Opisthosoma without ridges and furrows; rear prodorsal shield margin indistinct; tubercles of *sc* situated almost in middle of prodorsal shield. Associated with Rosaceae----------------------------------------------------------------------------------***Fragariocoptes* Roivainen 1951**

### 3.2. Description of Nothofagicola n. g. (Phytoptidae: Sierraphytoptinae: Sierraphytoptini)

**Genus *Nothofagicola* n. g.**

**Diagnosis**. Prodorsal shield with paired setae *ve* and *sc*. All common to Eriophyoidea leg and opisthosomal seta present (including *c1* and *φ* I). Tubercles of *ve* displaced to epicoxal area and situated below anterolateral margin of prodorsal shield that extends anteriorly and forms the lateral margin of the frontal lobe. Frontal lobe subtriangular, anteriorly rounded. Opisthosoma dorsally flattened. Dorsal medial opisthosomal ridge absent. Dorsal lateral ridges present or indistinct. Distal margins of dorsal opisthosomal annuli form thin overlapping plates. Associated with *Nothofagus* spp.

**Figure 1:**
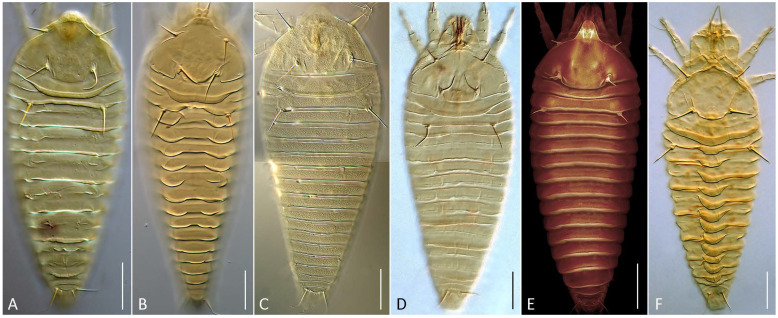
DIC LM (**A**–**D**,**F**) and CLSM (**E**) images of the mites of the genera *Nothofagicola* **n. g.**, *Sierraphytoptus* Keifer 1939 and *Solenocristus* Chetverikov et al. 2016 (dorsal aspect). (**A**)—*N. alpinae* **n. sp.**, (**B**)—*N. bicristata* **n. sp.**, (**C**)—*N. licanteniensis* **n. sp.**, (**D**)—*N. nothofagalis* **n. comb.**, (**E**)—*Si. alnivagrans*, (**F**)—*So. searsius*. Scale bar—25 μm.

**Differential diagnosis.** The new genus is close to *Solenocristus* and *Sierraphytoptus* (Figure 1). These genera differ in presence/absence of medial opisthosomal ridge (present only in *Solenocristus*) and tibial solenidion *φ* I (absent only in *Sierraphytoptus*). It is also close morphologically to deutogyne form of *Austracus*; however, correct separation needs additional investigation of the species diversity and the life cycle of *Austracus* [5].

**Etymology.** The new generic name, *Nothofagicola*, is derived from *Nothofagus*, the genus of the host plants, and suffix “-icola”, meaning “associated with”. Gender feminine.

**Type species:** *Nothofagicola alpinae* **n. sp.**

**Species included:** *N. alpinae* **n. sp.**, *N. bicristata* **n. sp.**, *N. licanteniensis* **n. sp.**, *N. nothofagalis* (Chetverikov 2020) **n. comb.** (transferred from *Solenocristus*).

**Remarks.** The species “*nothofagalis*” was originally described as a member of *Solenocristus* Chetverikov et al. 2016 under the name *Solenocristus nothofagalis* Chetverikov 2020. This species does not have a median opisthosomal ridge. We exclude it from *Solenocristus* Chetverikov et al. 2016 and transfer to *Nothofagicola* **n. g.**

**Hosts.** *Nothofagus* spp. (Nothofagaceae).

**Relation to host:** Vagrant on leaves and stems causing no visible damage to hosts.

**Distribution.** Currently known only from South America (Argentina and Chile).

### 3.3. New Species and Records of Nothofagicola n. g. from Chile

**Figure 2:**
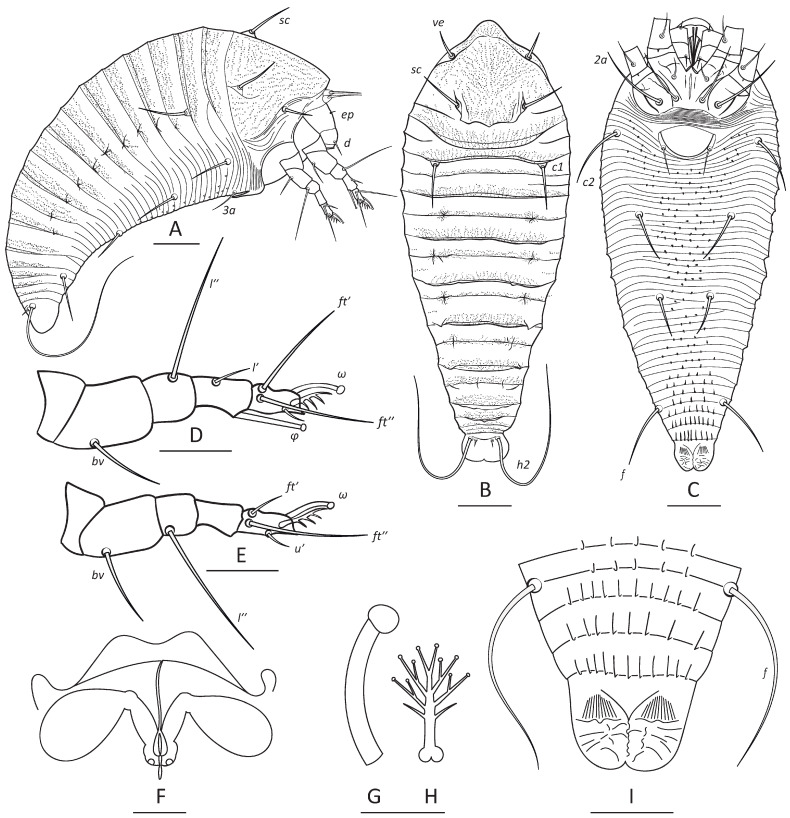
Drawings of *N. alpinae* **n. sp.** (female). (**A**–**C**)—lateral (**A**), dorsal (**B**) and ventral (**C**) view of entire mite; (**D**)—leg I, (**E**)—leg II, (**F**)—internal genitalia, (**G**)—tarsal solenidion I, (**H**)—empodium I, (**I**)—ventral view of telosoma. Scale bar: (**A**–**C**)—20 µm; (**D**,**E**,**I**)—10 µm; (**F**)—5 µm; (**G**,**H**)—3 µm.

**Figure 3:**
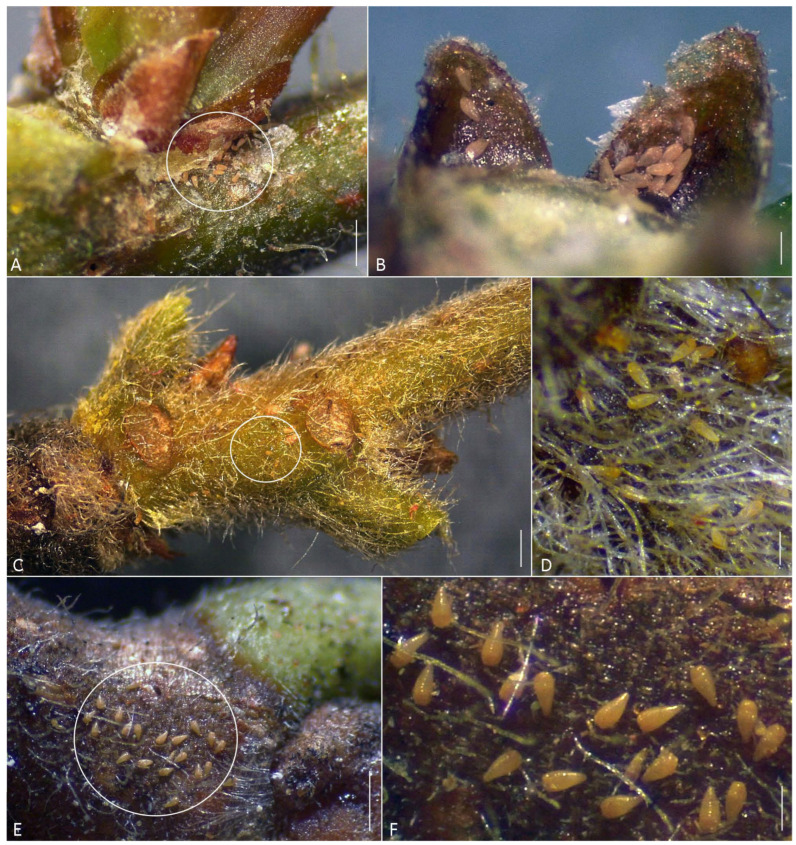
Groups of live mites of the genus *Nothofagicola* **n. g.** on shoots of *Nothofagus*. (**A**,**B**)—*N. alpinae* **n. sp.** near lateral bud (**A**), (white ellipse) and under bud scale (**B**) of *No. alpina*; (**C**) (white ellipse) and (**D**)—*N. bicristata* **n. sp.** among hairs on the basal young stem of *No. alessandrii*; (**E**) (white ellipse) and (**F**) (enlarged (**E**))—a group of *N. licanteniensis* **n. sp.** near the base of young stem of *No. glauca*. Scale bar: (**A**,**C**,**E**)—1 mm; (**B**,**D**,**F**)—0.25 mm.

***Nothofagicola alpinae* n. sp.**—Figure 1A, Figure 2 and Figure 3A,B.

**HOLOTYPE AND PARATYPE FEMALES** (*n* = 10). Body flattened, orange to brownish, 191 (177–195), 72 (72–75) wide at the level of setae *c2*. Prodorsal shield subtriangular, 46 (43–46) including frontal lobe, 65 (63–66) wide. Frontal lobe 9 (8–11), 20 (18–20) wide, subtriangular, rounded anteriorly, smooth ventrally. Prodorsal shield lines or ridges absent, cuticle of prodorsal shield with numerous micropores or microgranulations. Prodorsal shield setae *ve* and *sc* stout, approximately 1 µm thick: *ve* 13 (11–13), directed anterolaterad, tubercles of *ve* situated slightly below anterolateral margin of prodorsal shield, 32 (31–33) apart; *sc* 18 (17–20), 28 (26–29) apart, directed up and divergently anteriad. Distance between tubercles of *ve* and *sc* 28 (26–28). Epicoxal area with 7–10 thin smooth subparallel ridges.

**Gnathosoma** directed obliquely down and slightly forward. Palps 22 (22–25); chelicerae 16 (16–18). Gnathosomal setae: seta *ν* 2 (1–2); pedipalp genual seta *d* non-bifurcate, 7 (5–7); pedipalp coxal seta *ep* 3 (2–4). Suboral plate (*n* = 6) subtriangular, with two diverging ridges forming wide V-like shape, 10 (9–10), 19 (17–20) wide.

**Leg I** 36 (32–36), tarsus 6 (6–7), *u*’ 3 (3–4), *ft*’ 18 (17–20), *ft*″ 20 (19–23), tarsal solenidion *ω* 6 (6–7) knobbed; empodium 5 (5–6), symmetrical, 4-rayed; tibia 7 (6–8), *l*’ 7 (6─7); tibial solenidion *φ* 9 (9─10) knobbed; genu 5 (5–6), *l*″ 15 (15–20), located on dorsal surface of genu I; femur 12 (12–13), *bv* 10 (8–11).

**Leg II** 30 (29–32), tarsus 6 (6–7), *u*’ 3 (2–4), *ft*’ 4 (4–6), *ft*″ 18 (18–20), *ω* 7 (6–7) knobbed; empodium 6 (5–6), symmetrical, 4-rayed; tibia 6 (5–6); genu 5 (4–6), *l*″ 20 (20–21), located on lateral surface of genu close to femurogenual articulation; femur 10 (10–11), *bv* 11 (9–12).

**Coxal plates** almost smooth, with few faint irregular cuticular ridges and distinct outlines of coxal apodemes; prosternal apodeme 12 (10–12), flanked by two arc-shaped ridges, *1b* 9 (7–9), 13 (13–14) apart; *1a* 19 (16–20), 9 (9–10) apart; *2a* 24 (24–30), 22 (22–23) apart; 10 (10–15) narrow coxigenital semiannuli bearing tiny rounded microtubercles before epigynium.

**External genitalia.** Genital coverflap rounded, smooth, 14 (13–14), 20 (19–21), wide; setae *3a* 11 (10–13), 18 (18–20) apart; pregenital plate absent.

**Internal genitalia (*n* = 5).** Spermatheca ovoid, 7–9 long, 5–7 wide, directed latero-posteriad; spermathecal tubes 6–8 long, 2–3 wide, directed latero-anteriad; spermathecal process absent; longitudinal bridge 11–13 with rudimentary postspermathecal part; anterior genital apodeme subtrapezoidal; oblique apodeme absent.

**Opisthosoma** dorsally with 17 (17–18) semiannuli, ventrally with 51 (46–51) microtuberculate semiannuli. Dorsal semiannuli 1, 2, 4, 6, 8, 10, 12, 14, and 15 form thin distal plates. Dorsal plate of the 1st dorsal annulus rounded, entire, flanked laterally by two incomplete lateral semiannuli inserted laterally between 1st and 2nd dorsal opisthosomal annuli. Microtubercles on ventral annuli present mostly midway in the area delimited by tubercles of opisthosomal setae, they are rounded behind external genitalia, becoming larger and pointed beyond setae *e*, and more elongated and ridge-like on ventral annuli beyond setae *f*. Setal lengths: *c1* 19 (18–20), *c2* 14 (14–17), *d* 22 (18–22), *e* 19 (18–20), *f* 25 (24–28); *h1* 6 (4–6); *h2* 65 (53–65); 2* dorsal semiannuli from rear shield margin to *c1*; 5 (4–6) ventral semiannuli between external genitalia and tubercles of *c2*; 12 (10–12) semiannuli between *c2* and *d*; 14 (12–15) semiannuli between *d* and *e*; 13 (12–14) semiannuli between *e* and *f*; and 3 (3–4) semiannuli between *f* and *h2*.

**MALE.** Not found.

**GenBank data.** PZ161092 (*COI*), PZ161093 (*D1D2 28S*).

**Host plant.** *Nothofagus alpina* Popp. & Endl. (Nothofagaceae).

**Relation to the host plant.** The most mites were found feeding on leaf petioles or among hairs on young stems. Groups of 5–12 motionless mites were also observed under bud scales (Figure 3A,B). No visible damage to host was observed.

**Type material.** Type female in slide v2051-2, 13 paratype females in slide series v2050-1, v2051-1, v2052-1 collected in CHILE: about 50 km south-east of Chillan, 36°55′28.4″ S 71°34′08.9″ W, 13 March 2025, coll. P. Chetverikov and L. Peralta. Type slides and ethanol material are kept in Acarological collection of ZIN RAS and in SAG, Curicó (Chile).

**Etymology**. The species name is a noun in the genitive case, gender feminine, derived from the species name of the host plant “*alpina*” (adjective).

**Differential diagnosis.** The new species is closest to *N. bicristata* **n. sp.** (described below). In *N. alpinae* **n. sp.** the paired plates of dorsal opisthosomal annuli are absent (present and mark lateral opisthosomal ridges in *N. bicristata* **n. sp.**) and the wide plate formed by the 1st dorsal annulus is entire (with short median ridge in *N. bicristata* **n. sp.**).

 

**Figure 4:**
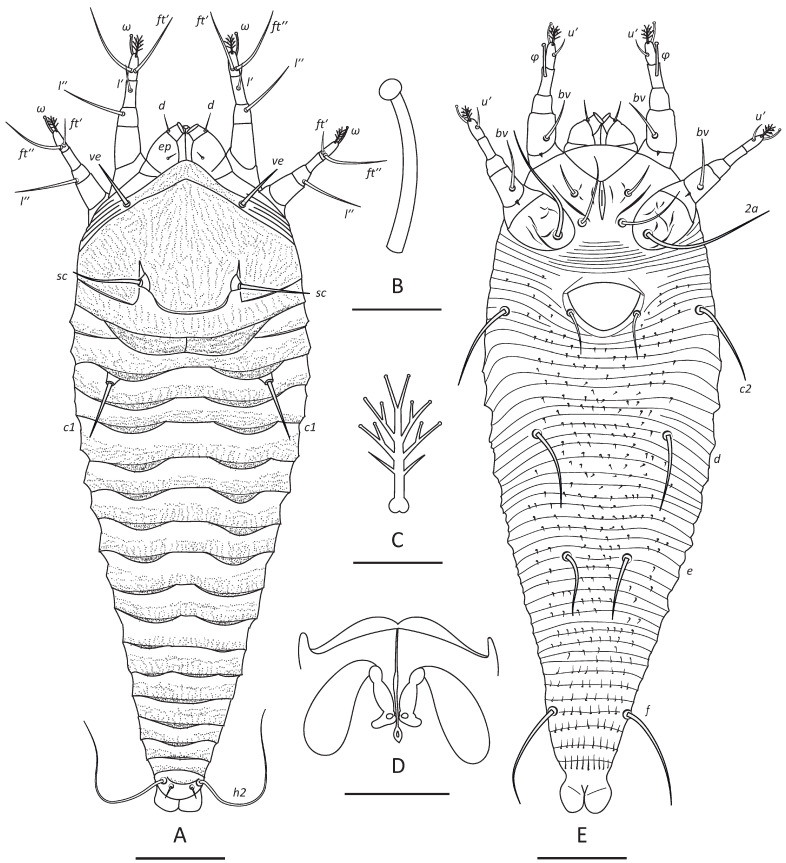
Drawings of *N. bicristata* **n. sp.** (female). (**A**)—dorsal view of entire mite, (**B**)—tarsal solenidion I, (**C**)—empodium I, (**D**)—internal genitalia, (**E**)—ventral view of entire mite. Scale bar: (**A**,**E**)—25 µm; (**B**,**D**) = 3 µm; (**D**)—5 µm.

***Nothofagicola bicristata* n. sp.—**Figure 1B, Figure 3C,D and Figure 4.

**HOLOTYPE AND PARATYPE FEMALES (n = 12).** Body flattened, dark orange to brownish, 208 (193–216), 69 (69–74) wide at the level of setae *c2*. **Prodorsal shield** subtriangular, 43 (43–46) including frontal lobe, 64 (64–69) wide. Frontal lobe 5 (4–6), 18 (18–20) wide, subtriangular, rounded anteriorly, with two subparallel ventral ridges. Prodorsal shield lines or ridges absent, cuticle of prodorsal shield with numerous micropores or microgranulations. Prodorsal shield setae *ve* and *sc* stout and approximately 1 µm thick: *ve* 12 (12–15), directed anterolaterad, tubercles of *ve* situated slightly below anterolateral margin of prodorsal shield, 34 (33–34) apart; *sc* 17 (17–20), 25 (24–28) apart, directed up and divergently anteriad. Distance between tubercles of *ve* and *sc* 23 (22–24). Epicoxal area with 6–8 thin smooth subparallel ridges.

**Gnathosoma** directed obliquely down and slightly forward. Palps 22 (20–23); chelicerae 16 (15–17). Gnathosomal setae: seta *ν* 1 (1–2); pedipalp genual seta *d* non-bifurcate, 5 (5–8); pedipalp coxal seta *ep* 3 (3–4). Suboral plate subtriangular, smooth (n = 5), 9 (9–10), 21 (20–22) wide.

**Leg I** 35 (35–37), tarsus 7 (6–8), *u*’ 5 (4–5), *ft*’ 16 (16–20), *ft*″ 21 (21–23), tarsal solenidion *ω* 8 (7–8) knobbed; empodium 6 (5–6), symmetrical, 4-rayed; tibia 8 (8–9), *l*’ 6 (6–8); tibial solenidion *φ* 11 (9–11) knobbed; genu 5 (5–6), *l*″ 15 (15–18), located on dorsal surface of genu I; femur 12 (12–13), *bv* 11 (11–13).

**Leg II** 30 (30–34), tarsus 7 (7–8), *u*’ 4 (3–4), *ft*’ 5 (4–6), *ft*″ 20 (18–21), *ω* 7 (7–8) knobbed; empodium 6 (5–7), symmetrical, 4-rayed; tibia 6 (6–7); genu 5 (4–5), *l*″ 19 (17–21), located on lateral surface of genu close to femurogenual articulation; femur 10 (10–12), *bv* 12 (10–13).

**Coxal plates** smooth with distinct outlines of coxal apodemes; prosternal apodeme 10 (10–12), flanked by two short subparallel ridges, *1b* 8 (8–10), 16 (15–16) apart; *1a* 18 (16–21), 12 (11–13) apart; *2a* 32 (30–38), 27 (25–28) apart; 15 (11–16) narrow smooth coxigenital semiannuli before epigynium.

**External genitalia.** Genital coverflap rounded, smooth, 13 (12–13), 22 (22–24), wide; setae *3a* 11 (11–14), 19 (19–21) apart; pregenital plate absent.

**Internal genitalia (*n* = 3).** Spermatheca tear-drop shaped, 8–11 long, 4–5 wide, directed latero-posteriad; spermathecal tubes 6–8 long, 2–3 wide, directed latero-anteriad; spermathecal process absent; longitudinal bridge 11–13 with rudimentary postspermathecal part; anterior genital apodeme subtrapezoidal; oblique apodeme absent.

**Opisthosoma** dorsally with 17 (17–18) semiannuli each with two thin distal plates forming together two lateral opisthosomal ridges separated by shallow median furrow, ventrally with 44 (44–46) microtuberculate semiannuli. First dorsal annulus forms rounded plate subdivided into two symmetrical parts by medial indentation and thin longitudinal ridge. Two incomplete lateral semiannuli inserted between 1st and 2nd dorsal opisthosomal annuli. Microtubercles of ventral annuli present mostly midway in the area delimited by tubercles of opisthosomal setae; they are smaller and spine-shaped in coxigenital area, becoming slightly larger beyond setae *e*, and more elongated and ridge-like on ventral annuli beyond setae *f*. Setal lengths: *c1* 18 (18–22), *c2* 19 (17–20), *d* 20 (17–20), *e* 17 (16–17), *f* 27 (26–30); *h1* 5 (4–5); *h2* 68 (60–69); 2* dorsal semiannuli from rear shield margin to *c1*; 5 (4–5) ventral semiannuli between external genitalia and tubercles of *c2*; 10 (10–12) semiannuli between *c2* and *d*; 12 (11–13) semiannuli between *d* and *e*; 14 (13–15) semiannuli between *e* and *f*; and 3* semiannuli between *f* and *h2*.

**MALE.** Not found.

**Genbank data.** PZ161090, PZ161091 (D1D2 *28S*).

**Host plant.** *Nothofagus alessandrii* Espinosa (Nothofagaceae).

**Relation to the host plant.** The mites were found feeding on leaf petioles and among hairs on young stems (Figure 1C,D). No visible damages to host were observed.

**Type material.** Type female in slide v2032-1, 22 paratype females in slides v2026-1 and v2027A-1 collected in CHILE: about 25 km north-west of Cauqenes, Reserva Nacional Los Ruiles, 35°50′00.9″ S 72°30′33.8″ W, 10 March 2025, coll. P. Chetverikov and L. Peralta. Type slides and ethanol material are kept in Acarological collection of ZIN RAS and in SAG, Curicó (Chile).

**Remarks.** Sampling in the Reserva Nacional Los Ruiles was conducted with authorization from the Regional Directorate of CONAF in the Maule Region, Chile.

**Etymology.** The species epithet *bicristata* is a feminine adjective derived from the Latin prefix “bi” (two) and “crista” (ridge). It refers to the pair of lateral ridges formed by the dorsal opisthosomal annuli.

**Differential diagnosis.** This species is closest to *N. alpinae* (see above).

 

**Figure 5:**
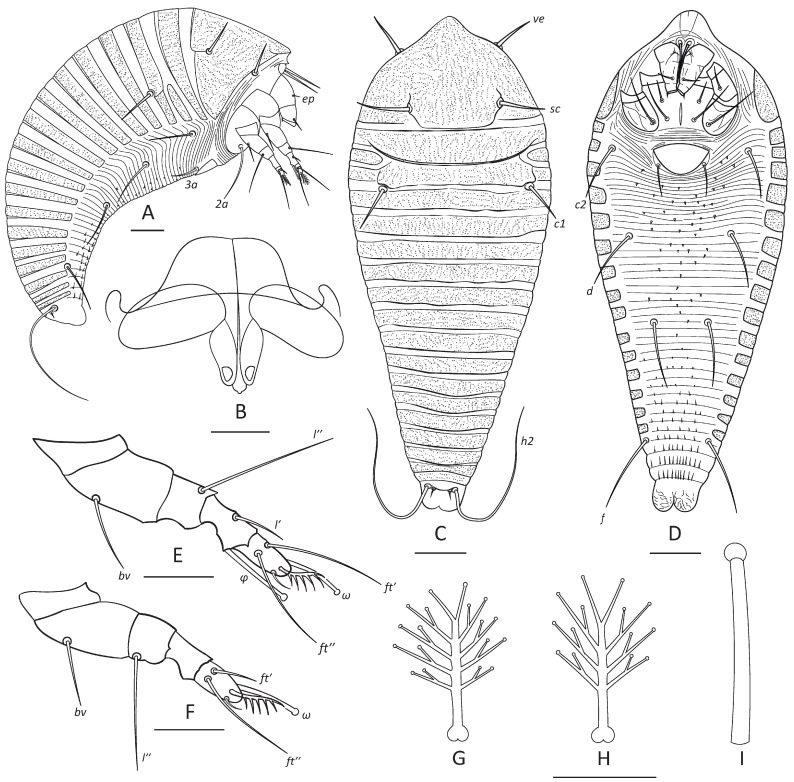
Drawings of *N. licanteniensis* **n. sp.** (female). (**A**,**C**,**D**)—lateral (**A**), dorsal (**C**) and ventral (**D**) view of entire mite; (**B**)—internal genitalia, (**E**)—leg I, (**F**)—leg II, (**G**)—5-rayed empodium, (**H**)—4-rayed empodium, (**I**)—tarsal solenidion I. Scale bar: (**A**,**C**,**D**)—20 µm; (**B**,**G**,**H**,**I**)—5 µm; (**E**,**F**)—10 µm.

***Nothofagicola licanteniensis* n. sp.—**Figure 1C, Figure 3E,F and Figure 5.

**HOLOTYPE AND PARATYPE FEMALES (*n* = 10).** Body flattened, yellowish to brownish, 205 (185–220), 83 (74–83) wide at the level of setae *c2*. **Prodorsal shield** subtriangular, 46 (45–52) including frontal lobe, 75 (68–75) wide. Frontal lobe 9 (8–10), 22 (18–22) wide, subtriangular, rounded anteriorly, with two ridges on ventral surface forming distinct V- or U-shaped figure. Prodorsal shield lines or ridges absent, cuticle of prodorsal shield with numerous micropores or microgranulations. Prodorsal shield setae *ve* and *sc* thick and stout, approximately 1 µm thick: *ve* 17 (14–17), directed anterolaterad, tubercles of *ve* situated slightly below anterolateral margin of prodorsal shield, 39 (36–39) apart; *sc* 19 (16–19), 37 (35–37) apart, directed up and divergently anteriad. Distance between tubercles of *ve* and *sc* 22 (22–23). Epicoxal area with 6–10 thin smooth subparallel ridges.

**Gnathosoma** directed obliquely down and slightly forward. Palps 22 (21–23); chelicerae 16 (16–18). Gnathosomal setae: seta *ν* 1 (1–2); pedipalp genual seta *d* non-bifurcate, 5 (5–7); pedipalp coxal seta *ep* 2 (2–4). Suboral plate (n = 5) subtriangular, smooth, 10 (10–11), 20 (18–22) wide.

**Leg I** 37 (34–38), tarsus 8 (7–8), *u*’ 5 (5–7), *ft*’ 18 (18–22), *ft*″ 20 (20–24), tarsal solenidion *ω* 8 (8–10) knobbed; empodium 7 (7–8), symmetrical, 5-rayed, rarely 4-rayed; tibia 8 (7–8), *l*’ 7 (7–9); tibial solenidion *φ* 10 (10–11) knobbed; genu 5 (5–6), *l*″ 20 (20–22), located on dorsal surface of genu I; femur 12 (11–13), *bv* 14 (13–14).

**Leg II** 34 (31–34), tarsus 7 (6–7), *u*’ 5 (4–6), *ft*’ 5 (5–7), *ft*″ 20 (19–20), *ω* 9 (8–10) knobbed; empodium 8 (7–8), symmetrical, 5-rayed, rarely 4-rayed; tibia 7 (6–7); genu 5 (5–6), *l*″ 23 (21–24), located on lateral surface of genu close to femurogenual articulation; femur 10 (10–12), *bv* 15 (14–16).

**Coxal plates** with faint irregular cuticular ridges; prosternal apodeme 8 (7–12), *1b* 7 (6–9), 16 (14–16) apart; *1a* 15 (15–19), 10 (9–11) apart; *2a* 25 (20–30), 22 (22–23) apart; 7 (7–10) narrow smooth coxigenital semiannuli before epigynium.

**External genitalia.** Genital coverflap rounded, smooth, 14 (13–16), 22 (22–23), wide; setae *3a* 12 (11–15), 20 (19–20) apart; pregenital plate absent.

**Internal genitalia (*n* = 3).** Spermatheca ovoid, 7–9 long, 4–5 wide, directed latero-posteriad; spermathecal tubes 5–7 long, 2–3 wide, directed latero-anteriad; spermathecal process absent; longitudinal bridge 14–17 with rudimentary postspermathecal part; anterior genital apodeme subtrapezoidal; oblique apodeme absent.

**Opisthosoma** dorsally with 17 (17–18) semiannuli forming thin distal plates, ventrally with 51 (46–51) microtuberculate semiannuli. Cuticle between dorsal annuli forms interannular bands with numerous tiny pores. First dorsal annulus forms rounded plate flanked by two elongate plates inserted laterally between 1st and 2nd dorsal opisthosomal annuli. Microtubercles on ventral annuli present mostly midway in the area delimited by tubercles of opisthosomal setae; they are rounded in coxigenital area, becoming larger and pointed beyond setae *e*, and more elongated and ridge-like on ventral annuli beyond setae *f*. Setal lengths: *c1* 18 (17–19), *c2* 20 (19–23), *d* 23 (19–25), *e* 27 (19–27), *f* 33 (30–33); *h1* 4 (4–6); *h2* 40 (38–63); 2* (dorsal semiannuli from rear shield margin to *c1*; 7 (5–8) ventral semiannuli from rear shield margin to *c2*; 13 (10–13) semiannuli between *c2* and *d*; 12 (11–16) semiannuli between *d* and *e*; 15 (13–15) semiannuli between *e* and *f*; and 4 (3–4) semiannuli between *f* and *h2*.

**MALE.** Not found.

**GenBank data.** PZ159406 (*COI*), PZ161089 (*D1D2 28S*).

**Host plant.** *Nothofagus glauca* (Phil.) Krasser (Nothofagaceae).

**Relation to the host plant.** Mites were found feeding on leaf petioles, among hairs on young shoot and near buds (Figure 3E,F). No visible damage to host was observed.

**Type material.** Type female in slide v1993-2, ten paratype females in slides v1993-1,3,4 collected in Chile: near Licantén, 34°57′35.0″ S 72°00′03.7″ W, along forest road, 3 March 2025, coll. P. Chetverikov and L. Peralta. Type slides and ethanol material are kept in Acarological collection of ZIN RAS and in SAG, Curicó (Chile).

**Etymology**. The species name *licanteniensis* is an adjective, gender feminine, derived from Licantén, the name of the town near the type’s locality.

**Differential diagnosis.** The new species is close to *N. alpinae* **n. sp.** (described above). These two species differ in general topography of dorsal opisthosoma (arched in *N. licanteniensis* **n. sp.** and flattened in *N. alpinae* **n. sp.**), in the presence/absence of lateral opisthosomal ridges (two weak ridges are present in *N. alpinae* **n. sp.** and absent in *N. licanteniensis* **n. sp.**), and the position of gnathosoma. Contrary to *N. alpinae* **n. sp.**, in *N. licanteniensis* **n. sp.** basal gnathosoma and coxae are situated in the depression of the cuticle of ventral prosoma (somewhat similar to ixodid camerostome).

 

***Nothofagicola* cf *nothofagalis* (Chetverikov 2020) n. comb.**

*Solenocristus nothofagalis* Chetverikov et al. 2020: 965, Figure 1, Figure 2, Figure 3 and Figure 4.

**Remarks**. This species was originally described based on the material from Argentina: Los Glaciares National Park, 49°18′57.5″ S 72°56′57.3″ W, along forest road, 13 December 2019, coll. A. Kiselev, lower leaf surface of *Nothofagus pumilio* (Poepp. & Endl.) Krasser (Nothofagaceae), no visible damage to host.

**New material from Chile.** In the end of February 2025 three populations (1, 2 and 3) of *N.* cf *nothofagalis* were collected in Southern Chile.

Population 1. Chile: island Chiloe, 42°12′32.0″ S 73°42′29.6″ W, 23 February 2025, 14 females and 5 males of *N.* cf *nothofagalis*, vagrant on the lower leaf surface of *Nothofagus pumilio* (Poepp. & Endl.), no visible damage to host, coll. C. A. Perez.

Population 2. Chile: about 20 km east of Puerto Natales, alongside road #9, 51°42′31.2″ S 72°18′56.3″ W, 19 February 2025, 12 females and 3 males of *N.* cf *nothofagalis*, vagrant on the lower leaf surface of *Nothofagus antarctica* (Forster) Oerst., no visible damage to host, coll. C.H. Alvarado.

Population 3. Chile: about 10 km east of Puerto Natales, alongside road #9, 51°41′28.5″ S 72°23′59.1″ W, 19 February 2025, 11 females and 2 males, vagrant on the lower leaf surface of *Nothofagus pumilio* (Poepp. & Endl.), no visible damage to host, coll. C.H. Alvarado.

**GenBank data.** Population 1: PZ159404 (*COI*), PZ161087 (*D1D2 28S*); population 2: PZ159405 (*COI*), PZ161088 (*D1D2 28S*).

### 3.4. Corrigendum

We found unfortunate typos in our previous paper on mitogenomics of the genus *Leipothrix* [23]. While all data from that paper submitted to GenBank are correct, in the published pdf file (https://doi.org/10.3390/insects14090759) and in the web version of the paper (https://www.mdpi.com/2075-4450/14/9/759, accessed on 16 April 2026), two incorrect combinations of the mite species and corresponding accession number of mitogenomic sequence are present. The correct accession numbers are given in Table 2.

## 4. Conclusions

South America is an underexplored continent for Eriophyoidea, offering significant potential for discovering new phytoptid diversity. In this paper, we established a new genus, *Nothofagicola* **n. g.**, to accommodate four phytoptid species from Chile and Argentina: *N. alpinae* **n. sp.**, *N. bicristata* **n. sp.**, *N. licanteniensis* **n. sp.** and *N. nothofagalis* **n. comb.** (transferred from *Solenocristus*). We also composed an updated dichotomous key to the world genera of the tribe Sierraphytoptini and incorporated all new sierraphytoptine genera described since the last world generic keys of Eriophyoidea were published in 2003. Along with another phytoptid genus, *Austracus* Keifer 1944, *Nothofagicola* **n. g.** shows specific associations with endemic *Nothofagus* hosts, some of which are endangered (*N. alpina*, *N. alessandrii*, *N. glauca*). This association may indicate the ancient biogeography of Phytoptidae s. str., as *Nothofagus* itself is a classic Gondwanan relict with a disjunct distribution across South America and Australasia. Since, to date, phytoptids have been recorded only on South American *Nothofagus* species, an important future direction is a systematic survey of phytoptids on *Nothofagus* in Australasia. Such surveys will determine whether this phytoptid lineage follows the classic Gondwanian disjunction—co-diversifying with its host across the entire former range—or whether it represents a more recently evolved, South America-endemic lineage.

## Figures and Tables

**Table 1:** Morphological traits separating genera of Sierraphytoptini (“+”—present, “−”—absent).

Morphological Traits	Genus								
	*Austracus* Keifr 1944	*Fragariocoptes* Roivainen 1951	*Neopropilus* Huang 1992	*Neoprothrix* (Reis and Navia 2014)	*Nothofagicola* n. sp.	*Prothrix* Keifer 1965	*Solenoplatilobus* Chetverikov & Craemer 2016	*Solenocristus* Chetverikov et al. 2018	*Sierraphytoptus* Keifer 1939
Tubercles of *ve* situated below anterolateral margin of prodorsal shield	−	+	+	+	+	+	+	+	+
Tibial seta *l*’ I	+	+	−	−	+	−	+	+	+
Seta *sc* (present/absent)	+	+	−	−	+	+	+	+	+
Tubercles of *sc* displaced to anterior part of prodorsal shield	−	−	−	−	−	+	−	−	−
Seta *c2*	+	+	−	+	+	+	+	+	+
Seta *d*	+	+	−	−	+	−	+	+	+
Seta *e*	+	+	−	−	+	+	+	+	+
Tibial solenidion *φ* I	+	−	+	+	+	+	+	+	−
Dorsal opisthosomal annuli forming distinct medial ridge	−	−	−	−	−	−	−	+	−
Frontal lobe of prodorsal shield	−	+	+	+	+	+	+	+	+
Telosomal pseudotagma	−	−	+	+	−	+	−	−	−

**Table 2:** Revised accession numbers of two mitogenomic sequences of *Leipothrix*.

Mite Species	Incorrect GenBank Accession Number Indicated in [23]	Correct GenBank Accession Number
*Leipothrix aegopodii* (Liro 1941)	OR268622	OR268621
*Leipothrix* cf *knautiae* (Liro 1942)	OR268621	OR268622

## Data Availability

All new DNA sequences obtained in this study have been deposited in the National Center for Biotechnology Information (NCBI) GenBank database (https://www.ncbi.nlm.nih.gov/genbank) (accessed on 17 March 2026).
